# IGRA tests perform similarly to TST but cause no adverse reactions: pediatric experience in Finland

**DOI:** 10.1186/1756-0500-2-9

**Published:** 2009-01-15

**Authors:** Esko Tavast, Eeva Salo, Ilkka Seppälä, Tamara Tuuminen

**Affiliations:** 1 Department of Bacteriology and Immunology, Haartman Institute, University of Helsinki, Helsinki, Finland; 2 Hospital for Children and Adolescents, University of Helsinki, Helsinki, Finland; 3 Division of Clinical Microbiology, Helsinki University Hospital, HUSLAB, Helsinki, Finland; 4 Department of Industrial Engineering and Management, Helsinki University of Technology, Helsinki, Finland

## Abstract

**Background:**

Two commercial interferon gamma release assays (IGRAs) (QuantiFERON^®^-TB Gold in Tube and T SPOT^®^-*TB*) to detect a contact with *M. tuberculosis *have recently become available. The majority of studies agree that the sensitivity and specificity of these methods are superior to the Tuberculin Skin Tests (TSTs) in detecting an exposure to bacteria in latently infected individuals and in clinical tuberculosis. However, the data in children remains limited.

**Findings:**

Consecutively collected samples from children (n = 99) representing age range from zero to 18 years were analyzed in a retrospective non-blinded study. The two IGRAs were modified and adapted to the needs of Finland, a country of a low tuberculosis incidence. For 27 children, both tests were performed simultaneously and compared with the TST and clinician's diagnosis. The sensitivity, specificity, and accuracy of both IGRAs was determined. QuantiFERON TB Gold and T SPOT-TB performed (respectively) as follows: sensitivities 0.92 (95% confidence interval, CI, 0.67–0.99) and 0.85 (0.64–0.95); specificities 0.91 (0.77–0.97) and 1.00 (0.93–1.00); accuracies 0.91 (0.80–0.97) and 0.96 (0.88–0.99). This compares favorably to the TST whose known figures are 0.90, 0.95, and 0.95, respectively. The agreement between the IGRAs was high, k = 0.89. Finally, both methods agreed well with the TST, k = 0.86 for TST/QuantiFERON-TB Gold and k = 0.76 for TST/T SPOT-TB.

**Conclusion:**

The sensitivity and specificity of IGRA methods compares well with the TST without the inconveniences and complications associated with TST, including exaggerated delayed type hypersensitivity reactions. These properties place them as acceptable substitutes for TST.

## Background

Diagnosis of childhood tuberculosis (TB) remains challenging [1]. Two commercial IFN-γ release assays (IGRAs), namely T-SPOT.*TB *and QuantiFERON^®^-TB Gold In-Tube (QFGT), have been recently developed. These methods show acceptable diagnostic accuracy for active TB in adults [2] and have better correlation with tuberculosis exposure than the TSTs in contact tracing for revealing latent infections (LTBI) [3,4]. However, pediatric data are limited, and studies evaluating the performance of IGRAs in children have been called for especially in low-incidence countries [2,5,6].

In Finland the incidence of TB is low, 5.6/100 000 inhabitants in 2006 [7]. Only zero to six child TB diagnoses per annum has been registered during the last ten years [8]. Universal BCG vaccination of newborns was practiced until 2006 [9]

Between September 2004 and January 2007 our laboratory adapted the tests to the general needs in Finland. After completion of the field evaluation [10] the methods became available for routine diagnostics. We then evaluated the performance of IGRAs in children and compared them to the TST and clinical diagnoses.

## Findings

### Study Population

**Figure 1:**
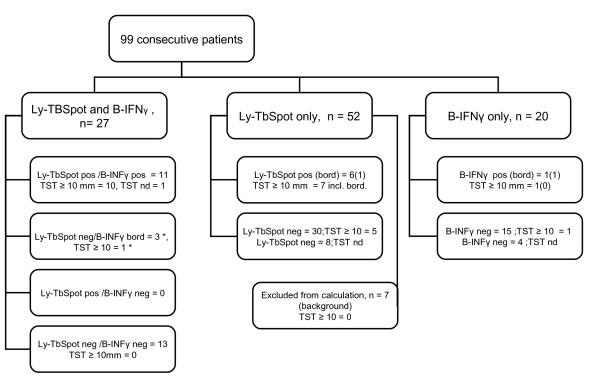
**The outline of the study design**. The (*) means that immunoconversion was observed in one patient. In this case the Ly-TbSpot, the *Mantoux *test and the B-TbIFNγ were done and in August and repeated in December, the two were negative and the B-TbIFNγ was once borderline but the repeated B-TbIFNγ test and the *Mantoux *turned positive in February (for more details see Table 3).

Between 15.9.2004 and 15.10.2007 we analyzed samples from 99 consecutive children and adolescents. The flow diagram of the study is presented in Fig. 1. We reviewed the medical records of the patients and collected data on demographics, clinical, microbiological and radiological examinations, and the BCG vaccination history (Table 1). This study was approved by the Ethical Committee of the Hospital for Children and Adolescents, University of Helsinki (Nr.164/E7/05).

**Table 1:** Characteristics of children enrolled in the study (n = 99)

Characteristic	n
Male	63

Age (yrs), median (min-max)	9 (0–18)

BCG vaccinated*	68
BCG non-vaccinated	6
BCG vaccination status unknown	25

Born outside Finland or at least one of the parents is from a country with endemic TB:	57
➢ Somalia (several families)	25
➢ Vietnam	6
➢ Peru (1 family)	4
➢ Russia	4
➢ Kosovo	2
➢ Nigeria	2
➢ Afghanistan, Angola, Azerbadzan, Burma Burundi, Estonia, India, Congo, Sudan, Thailand, Turkey, Uganda	12 (one each)
➢ Siblings who live in Russia	2

HIV infection	2

TST test performed	87
TST not performed	8
Data missing	4

Culture or/and Nucleic acid amplification (NAA) method performed	6
➢ acid fast staining positive	2/6 (also culture pos)
➢ culture positive (*M. tuberculosis*)	4/6
➢ NAA positive	2/6 (also culture pos)

Radiological examination performed	95
Radiological findings	
➢ normal	81/95 (85%)
➢ pulmonary TB	4
➢ enlargement of the hilus zone (Hodgkin lymphoma, TB lymphadenitis, one mild in LTBI, one mild in salmonelosis)	4 (one each)
➢ interstitial changes (vasculitis)	1
➢ pleural effusion (pleuritis and chylothorax)	2
➢ pneumonia	2
➢ bronchoectasia	1

Indications for IGRA-tests	
➢ exposure to TB	62
➢ clinical signs compatible with TB	37

* All children born in Finland before September 2006 were supposed to be BCG vaccinated even if this was not mentioned in the record.

### Ly-TbSpot procedure and quality control

The Ly-TbSpot, a modified version of T-SPOT.*TB *(Oxford Immunotec, Oxford, UK) [11], was performed using standard operation procedures (SOP). Firstly, the results were expressed as a number of reactive spots/million lymphocytes. The lymphocyte count from isolated peripheral blood mononuclear cell (PBMC) preparation was calculated with an automated hematologic analyzer (Advia^® ^60, Bayer, Germany). Secondly, duplicate ELISPOT wells were used for each antigen and media. Thirdly, Purified Protein Derivative (PPD) (Statens Serum Institut, Copenhagen, Denmark) was used as an additional positive control. Fourthly, we adopted a double cut-off policy, i.e. the responses below 25 spot/million lymphocytes were considered as non-reactive according to the guidelines by the manufacturer; the responses between 25 and 55 spots were considered borderline and over 55 spots were interpreted as reactive. An internal control of cryopreserved cells was run with each new batch of reagents.

### B-TbIFNγ procedure and quality control

**Figure 2:**
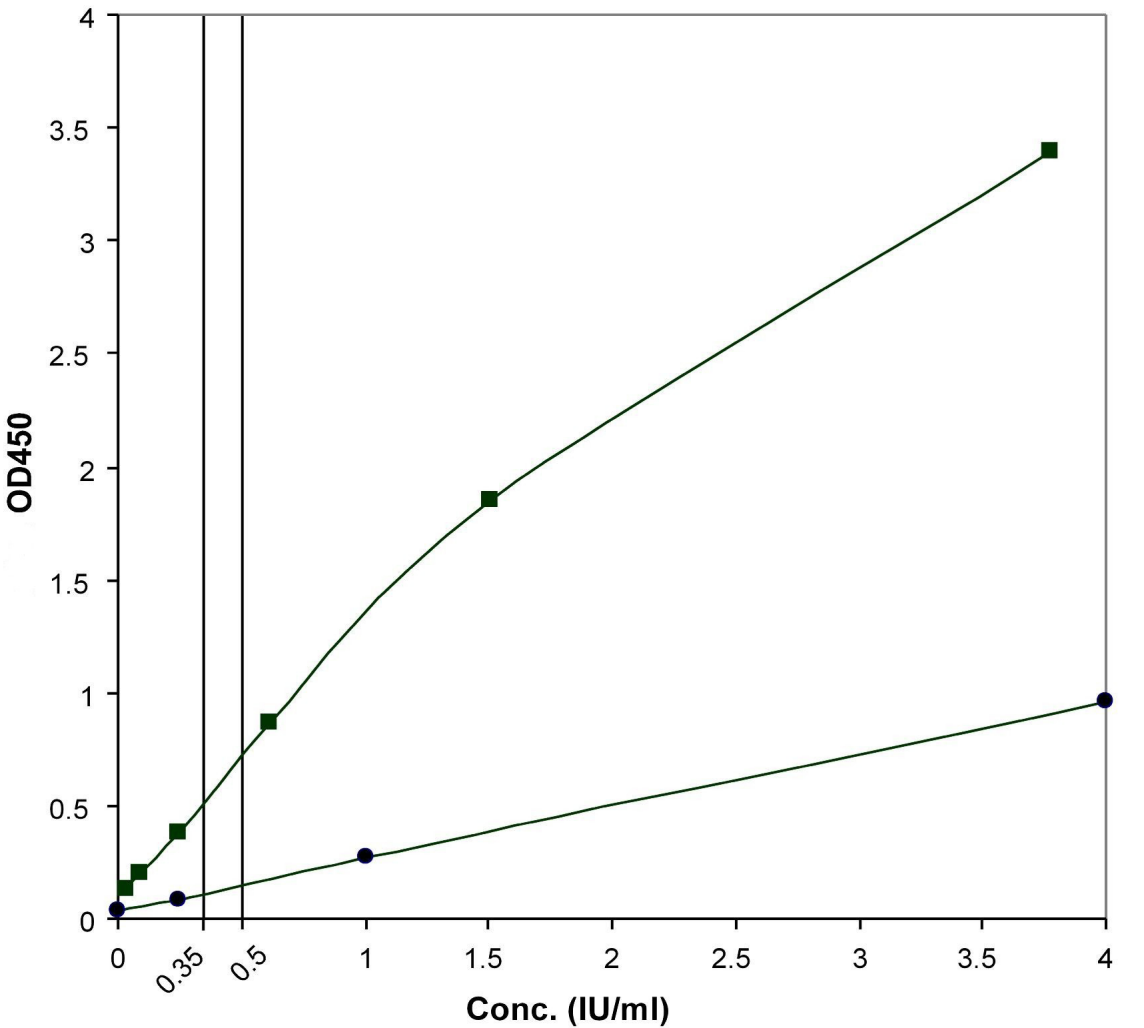
**Improvement of the calibration curve for IFNγ-measurement**. For measurement of IFN-γ levels, the original EIA reagent from Cellestis (circles) was substituted by that of PeliKine Compact human EIA (squares). This resulted in a steeper calibration curve and the use of the whole dynamic range of the photometer. Analytically, this means more accurate result interpretation in the cut-off zone. OD_405 nm_, optical densities measured at 405 nm.

B-TbIFNγ is a modified version of the QuantiFERON^®^-TB Gold In-Tube (Cellestis Limited, Carnegie, Victoria, Australia) [12]. In accordance with the SOP of our laboratory, stimulation of blood cells was done in tubes of the manufacturer. However, for measurement of IFN-γ levels, we used EIA of PeliKine Compact human EIA (Sanquin, Amsterdam, The Netherlands). This resulted in a steeper calibration curve and ensured more accurate result interpretation in the cut-off zone (Fig. 2). Two cut-off levels were applied. The samples showing a net reactivity (the reactivity of a sample minus the reactivity of the nil control) < 0.35 IU/ml were interpreted as non-reactive. Those showing reactivity between 0.35 and 0.50 IU/ml were interpreted as borderline and those with a reactivity exceeding 0.50 IU/ml were interpreted as reactive. For the internal quality control, an artificially prepared IFN-γ solution to simulate a low reactive sample was used.

### Method of TST

TST was performed with two TU, purified protein derivative (PPD, RT23, Statens Serum Institut, Copenhagen, Denmark) according to the *Mantoux *technique. The result was recorded after 48–72 h. A cut-off for positivity was defined as 10 mm or more in BCG-vaccinated and 5 mm or more in non-vaccinated children. If a child had a negative TST result, but was examined because of contact with an infectious TB case, the test was repeated [13].

### Definitions

For methods evaluation we used conventional interpretations of definitive and probable TB and of LTBI.

### Exclusion criteria

The results of the tests were considered eligible if the reactivities to the mitogen were as suggested by the manufactures. Seven samples were excluded from the ELISPOT analysis because of high non-specific background.

### Statistical analysis

The concordances between the IGRAs, and of each test with the TST, were assessed in three categories by describing proportions of agreement (PA) and with the Cohen's kappa statistic with linear weighting. 95% confidence intervals (CI) were calculated with the Wilson efficient-score method which was corrected for continuity. Using a conservative approach, the cases interpreted in the grey zone were placed into categories of false positives or false negatives for the calculations of specificity, sensitivity and accuracy.

### Final diagnosis of the participants

Overall, 99 samples were analyzed, and their characteristics are shown in Table 1, Table 2, and Figure 1. The subjects were tested as follows: with the Ly-TbSpot alone, with the B-TbINFγ alone, and with both Ly-TbSpot and B-TbIFNγ. Participant numbers were 52, 20, and 27 (out of 99 total), respectively. Seven results were excluded from calculations because non-specific background reactivity made it impossible to interpret the samples as definitely non-reactive. Of those tested with the Ly-TbSpot alone, six cases were positive and one was a borderline case; of those tested with the B-TbINFγ alone, one was interpreted as a borderline and one was positive; of those tested with both methods, 11 were positive by both methods and two samples were interpreted as borderline only with the B-TbINFγ method. The majority of the patients were either new immigrants or had at least one parent from a country with a high incidence of TB. The median age of the participants was nine years and the age range was from two weeks to adolescence.

**Table 2:** Final diagnoses of the patients

Diagnosis		Total number	TST performed, number	TST, mm median (range)
TB detected, n = 23	Pulmonary tuberculosis	5	5	16 (15–27)
	➢ 1 acid fast staining and culture pos			
	➢ 4 with radiologic findings, history of contact and positive TST			
				
	Extrapulmonary tuberculosis	5	4	18.5 (16–30)
	➢ 4 lymphadenitis (one HIV+), 3/4 culture positive			
	➢ 1 with clinical presentation and positive TST			
	
	LTBI	13	13	16 (10–27)

No TB detected, n = 76	Healthy contacts of TB cases	45	45	0 (0–9)
	
	Abscesses of diverse localizations	4	3	0 (0–4)
	
	Pneumonia or acute respiratory infection	5	5	0
	
	BCG osteitis	2	2	13.5 (12–15)
	
	Hypersedimentation due to obesity	2	2	0
	
	Cysticercosis	2	1	0
	
	Various infectious or inflammatory conditions, one each	16	9	0 (0–7)

The major indication for performing IGRA was recent contact with an infectious case of TB (62 of 99 patients). Thirty-seven children were examined because of symptomatic illness, with TB considered a diagnostic modality. Ten children were diagnosed with TB; five had pulmonary TB, only one of them culture positive; five were diagnosed with extrapulmonary TB. One patient also had an HIV infection, and one patient was HIV infected but had no TB.

Immunological conversion was observed in a 13-year-old boy who was examined several times because of the repeated household contacts with TB. His patient history and the kinetics of the immunological responses in all the three methods are presented in Table 3.

**Table 3:** Anamnestic data and the observed kinetics of immunological conversion in a 13-year-old boy.

Time	2007.07	2007.08	2007.09	2007.11	2007.12	2008.02
Anamnesis	A visitor in the family with pulmonary TB(AFB+)		The grandparent diagnosed with culture-positive TB			

TST (mm)		0 (neg)		16 (conversion)		

Ly-TbSpot (reactive cells/10^6 ^lymphocytes.		17 (non-reactive)		13 (non-reactive)	nd	nd
The max response is shown						

B-TbINFγ (IU/ml)		0.36 (borderline)		nd	0.15 (non-reactive)	0.70 (reactive)

**Figure 3:**
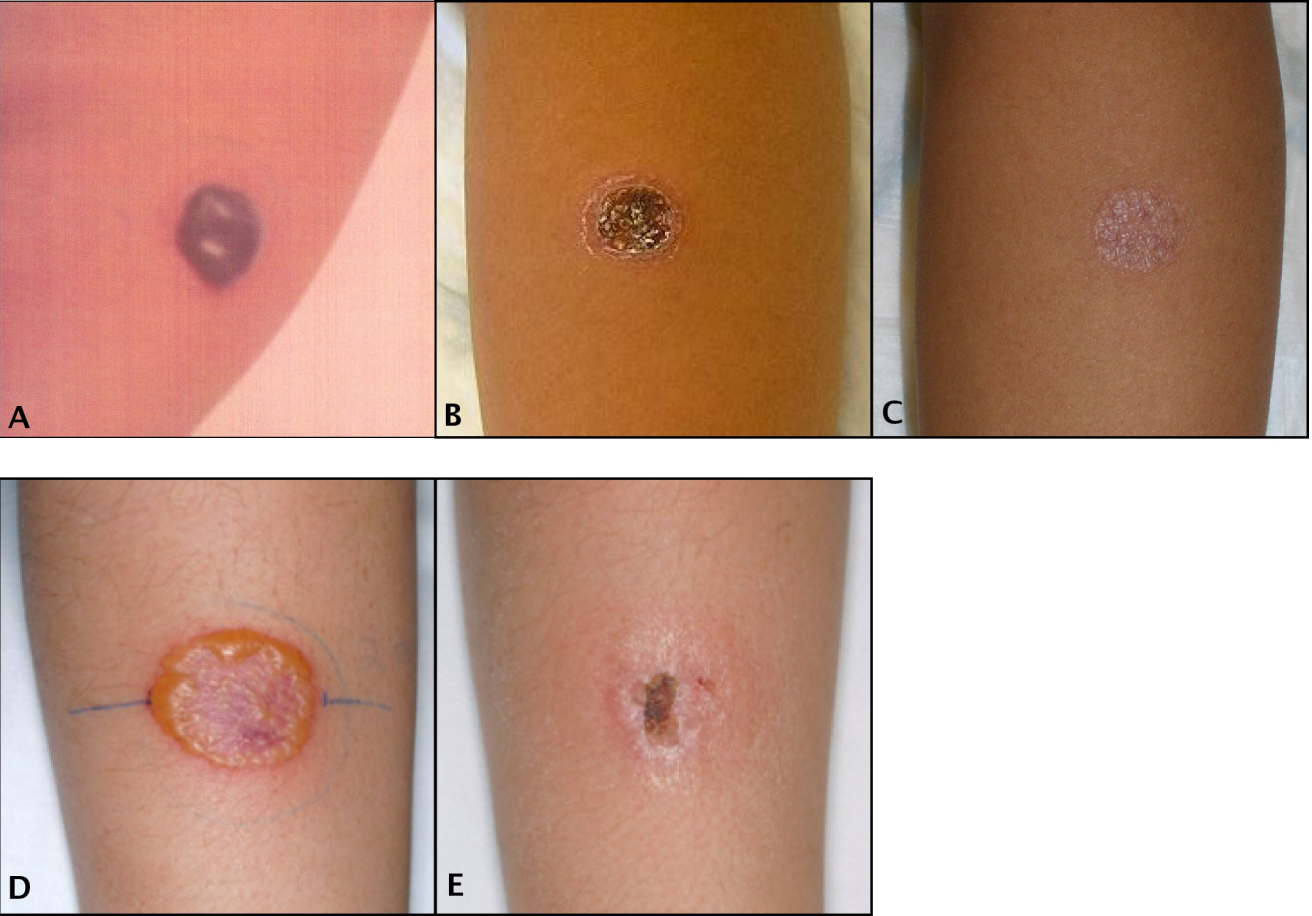
**Exaggerated delayed type hypersensitivity reaction in the TST leading to a permanent scar**. The upper panel represents results of the TST in a 14-year-old boy with LTBI, **A **taken three days, **B **two weeks and **C **three months after inoculation of 2 TU of PPD RT23. The original size of the induration was 20 mm. Photos: Courtesy of Dr. Peter Floman, Hospital of Porvoo. The lower panel represents results of the TST in a 13-year old girl with TB lymphadenitis, **D **taken 3 days and **E **two weeks after performing TST. This child was not enrolled in the current study.

The TST was performed in 87 (out of 99) subjects, in eight the test was not performed, and in four the data was missing from the records. Of those tested, positive TST was recorded in 25 cases, the size of the induration ranging from 10 to 30 mm. In one subject the TST converted from zero to 16 mm (Table 3). In another subject the TST test caused a severe delayed type hypersensitivity reaction, which resulted in a cosmetically unacceptable scar (Fig. 3A–C). A similar severe hypersensitivity reaction was observed in a third case that was not enrolled in the current study (Fig 3D, E).

### Performance characteristics of the IGRAs

Combined performance characteristics for IGRAs are presented in Table 4. None of the samples tested with the Ly-TbSpot were condemned as false positives, whereas three samples fell into the borderline category with the B-TbIFNγ (see Fig. 1). According to our interpretation criteria, three patients in the Ly-TbSpot and one in the B-TbIFNγ cohorts were grouped as false negatives. One was a 7-year-old boy with tuberculous lymphadenitis who had a borderline Ly-TbSpot result in our definition, but the TST induration was 20 mm. The second was a 15-year-old boy with TB contact who had TST induration of 13 mm but his Ly-TbSpot results were repeatedly negative. The third case was a 6-year-old girl with no symptoms whose sibling was diagnosed with TB. She had a TST conversion within a year, the TST induration at the time of assessment was 10 mm, but both IGRAs remained non-reactive. The fourth case was a 9-year-old boy from a highly endemic area whose TST induration was 11 mm but the B-TbIFNγ remained non-reactive. Because of the lack of the gold standard for LTBI, in the last three cases the diagnosis was based primarily on extensive exposure to TB and positive TST, in accordance with the recommendations in our country [13].

**Table 4:** Performance characteristics of the IGRA-methods.

Parameter	Sensitivity (95% CI^§^)	Specificity 95% CI)	PPV^† ^(95% CI)	NPV^‡ ^(95% CI)	Accuracy (95% CI)
Method					
**Ly-TbSpot**	0.85	1.00	1.00	0.96	0.96
n = 72	(0.64 – 0.95)	(0.93 – 1.00)	(0.82 – 1.00)	(0.87 – 0.99)	(0.88 – 0.99)

**B-TbIFNγ**	0.92	0.91	1.00	0.96	0.91
n = 41	(0.67 – 0.99)	(0.77 – 0.97)	(0.76 – 1.00)	(0.84 – 0.99)	(0.80 – 0.97)

* Pre-test probability, † Positive predictive value, ‡ Negative predictive value, §Confidence interval.

### Agreement between Ly-TbSpot, B-TbIFNγ and the TST

The calculated data are presented in Table 5. The κ values above 0.75 imply good agreement, more than might have occurred by chance.

**Table 5:** Agreement between Ly-TbSpot, B-TbIFNγ and the TST

Test pair		n	Agreementpos/neg	Proportions of agreement (95% CI)	κ (95% CI)
Ly-TbSpot	B-TbIFNγ	27	11/13	0.89 (0.70 – 0.97)	0.89 (0.77 – 1.00)

Ly-TbSpot	TST	62	16/39	0.89 (0.77 – 0.95)	0.76 (0.59 – 0.92)

B-TbIFNγ	TST	43	11/28	0.90 (0.77 – 0.97)	0.86 (0.72 – 1.00)

### Analysis of non-interpretable results

Non-specific background activation of cells for IFNγ production was observed in seven samples analyzed with the Ly-TbSpot. Of those, six samples were from members of a family newly immigrated from Ethiopia. These patients showed reactivity without stimulation with an added antigen, the size of the spots being very small. This non-specific reactivity was interpreted as the reactivity of other than effector T-cell populations, most probably of NK cells. All results obtained with the B-TbIFNγ-method were acceptable.

### The influence of the cut-off levels on performance characteristics of the Ly-TbSpot

**Figure 4:**
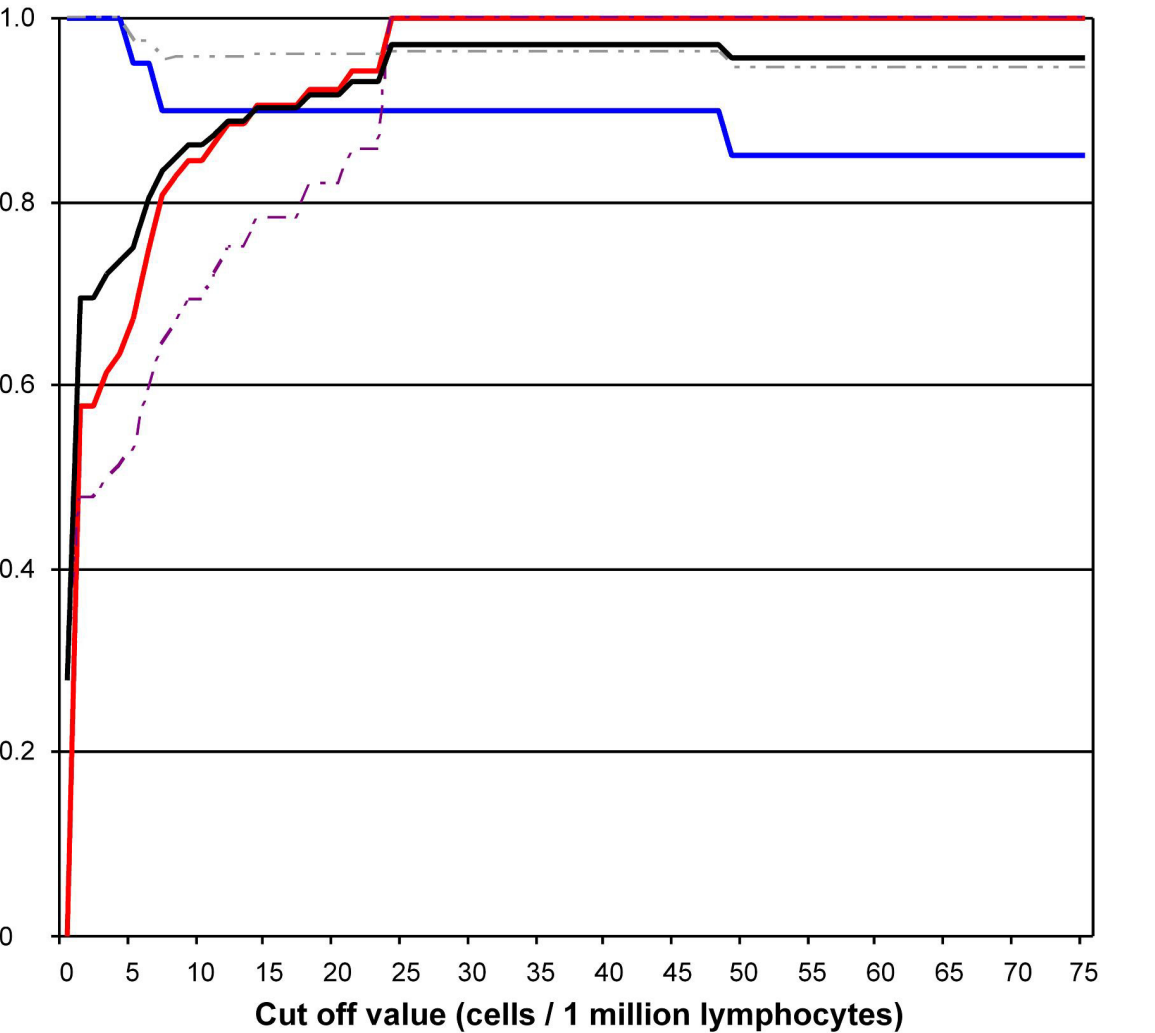
**Test parameters of Ly-TbSpot using differential cut-off points (n = 72)**. Theoretical values for sensitivity (blue), specificity (red), positive (dashed purple) and negative predictive values (dashed grey) on the y-axis were plotted against the cut-off points on the x-axis. The accuracy (black) of the tests peaked to 0.97 in the range of 24 to 48 spots/million lymphocytes. Hence, by preserving our current double cut-off policy (25 and 55 spots/million lymphocytes) we achieve an accuracy of 0.96.

Theoretical values for sensitivity, specificity, positive and negative predictive values were plotted against differential cut-off points. The analysis showed that the accuracy of the test peaked to 0.97 in the range of 24 to 48 spots/million lymphocytes (Fig. 4). Hence, by preserving our current double cut-off policy, and a conservative way of interpretation, we achieved an accuracy of 0.96. This means that any pediatric sample falling into the borderline category may most probably represent a pathological condition.

We present results of a retrospective, non-blinded study of two modified IGRAs for the diagnosis of childhood TB. The modifications were aimed to avoid false positive interpretations. Because no method can guarantee 100% sensitivity and specificity, we made a pragmatic decision to offer the best possible specificity even at the cost of sensitivity. Changing of the cut-off levels of the commercial IGRA methods has been suggested by other investigators. Lee at al [14] suggested lowering the cut-off in the QFGT method. This change would have resulted in a drop of specificity from 91.6% to 87.0% which we, contrary to Lee, do not regard as a minimal loss. Arend et al [15] also recommended dropping the cut-off of the QFGT to achieve a detection rate of potentially infected persons similar to that of TST. The recommendation is not acceptable for the reason that TST is by no means a gold standard. The sensitivity of QFGT could instead be improved e.g. by using more sensitive EIA techniques. In fact, although there was high agreement between IGRAs and TST in our studies, three earlier studies found a lower agreement: two studies [16,17] performed on pediatric samples in low risk countries observed concordant results between the IGRAs but inferior specificity of the TST, and one large cohort study of TB disease in African children [18] found lower agreement with the TST. In another cohort study of contact tracing, performed on Gambian children, the clinical sensitivity of the TST was found superior to that of the ELISPOT in diagnosing LTBI [19]. In that study, however, the discordance between the tests was not significant [19]. In a recent study from Gambia [20] comparing the new IGRAs to TST in contact tracing, ELISPOT was found more sensitive than the QFGT in the diagnosis of TB disease while equally sensitive in the diagnosis of LTBI. That study showed no significant discordance between IGRAs but the results of the TST were influenced by the BCG vaccination status. The observed discrepancies in the estimation of the three methods are most probably attributed to the differences in the research population, their vaccination status and exposure to other mycobacteria, and most importantly, in the variability of the methods' performance using variable threshold levels.

Our study raises several important issues. When performing TST, intradermal inoculation of PPD in children exposed to *M. tuberculosis *may produce an excessive delayed type hypersensitivity reaction. Secondly, demonstration of the test variability around the cut-off zone in real laboratory settings convinces that interpretation of immunodiagnostic methods should take into account the method imprecision and utilize a grey zone.

## Conclusion

The sensitivity and specificity of IGRA methods compares well with TST but do not cause exaggerated delayed type hypersensitivity reactions.

## Abbreviations

(TSTs): Tuberculin Skin Tests; (TU): Tuberculin Unit; (IGRA): Interferon Gamma Release Assays; (TB): Tuberculosis; (LTBI): Latent Tuberculosis Infection; (BCG): Bacille Calmette-Guérin; (SOP): Standard Operation procedure; Positive (PPV) and (NPV): Negative Predictive Values; (CI): Confidence Intervals; (PA): Proportions of Agreement; (ELISPOT): Enzyme-linked Immunosorbent Spot Assay; (Ly-TbSpot): Modified T-SPOT.*TB*; (ELISA): Enzyme-linked Immunosorbent Assay; (QFGT): QuantiFeronGold in Tube; (B-TbIFNγ): Modified QFGT; (NK): Natural Killer cells; (PHA): Phytohemagglutinin; (PPD): Purified Protein Derivative; (HIV): Human Immunodeficiency Virus.

## Competing interests

The authors declare that they have no competing interests.

## Authors' contributions

ET carried out statistical analysis and interpretation of the data and was involved in the drafting of the manuscript; TT and IS have contributed to the conception and design of the study, drafting and revision of the manuscript; ES is a clinician who treated presented cases, she contributed to the acquisition of the data and drafting of the manuscript. All authors have given final approval of the version to be published.
